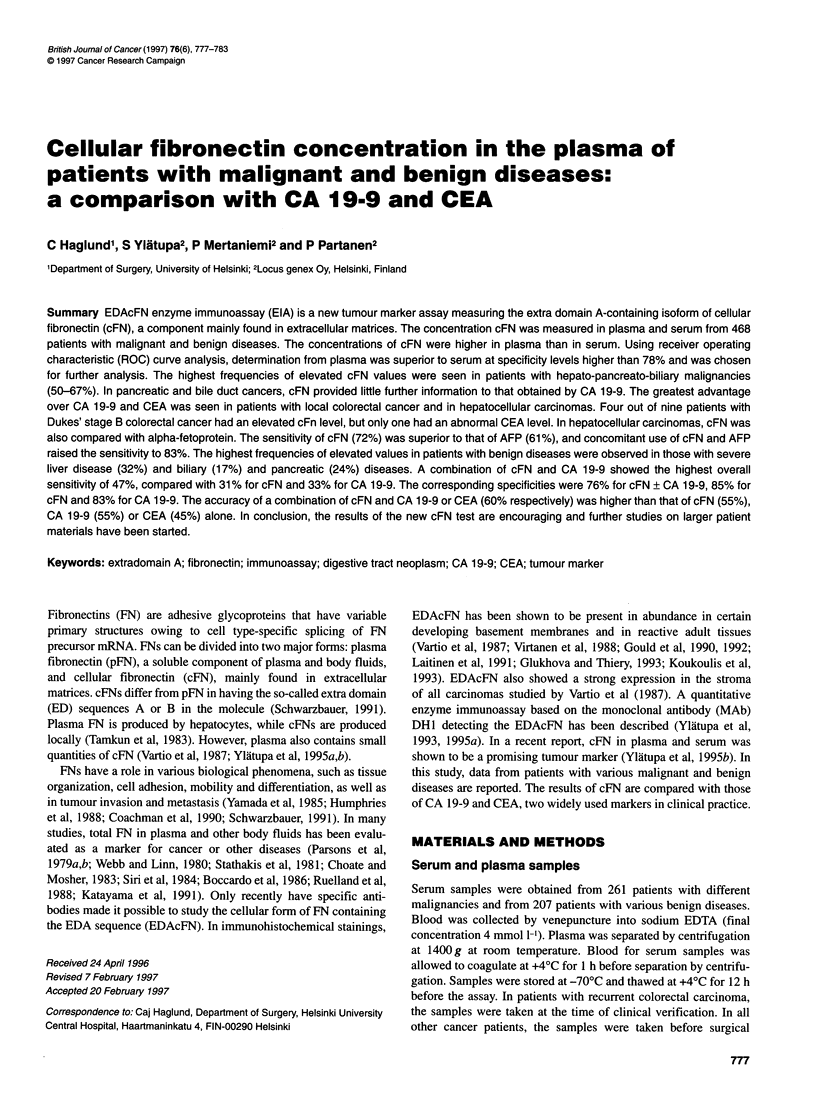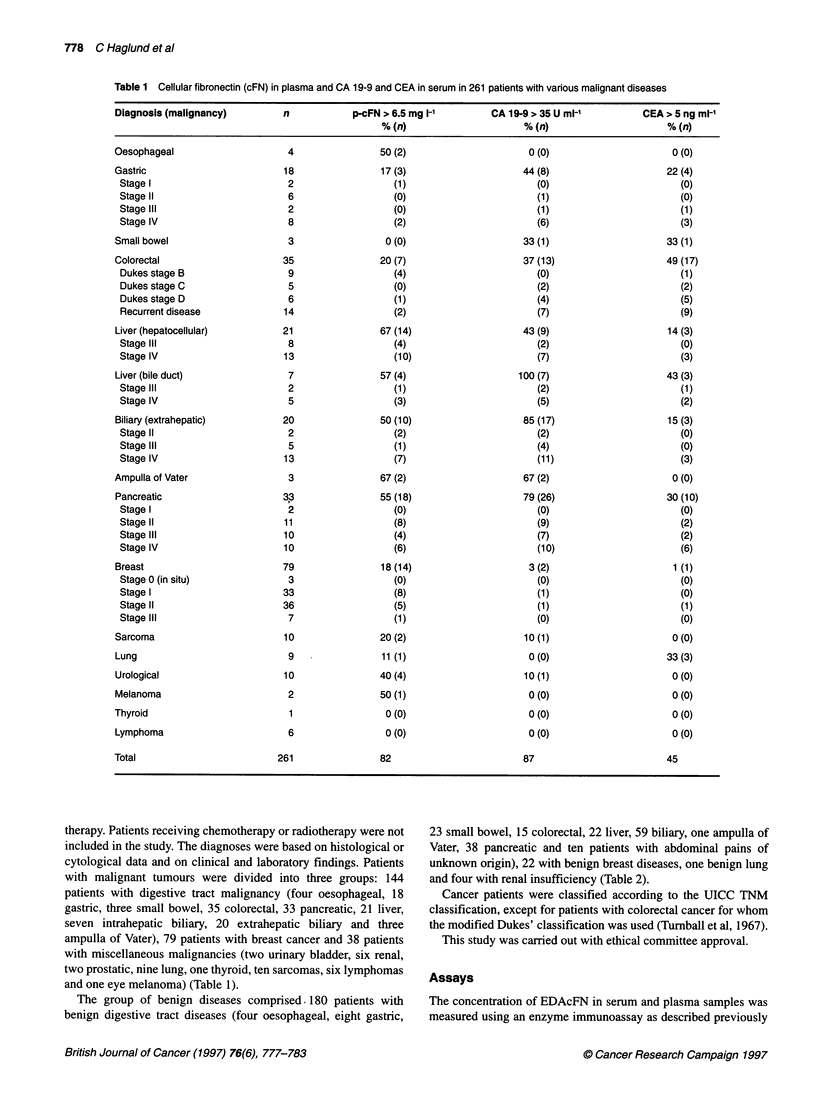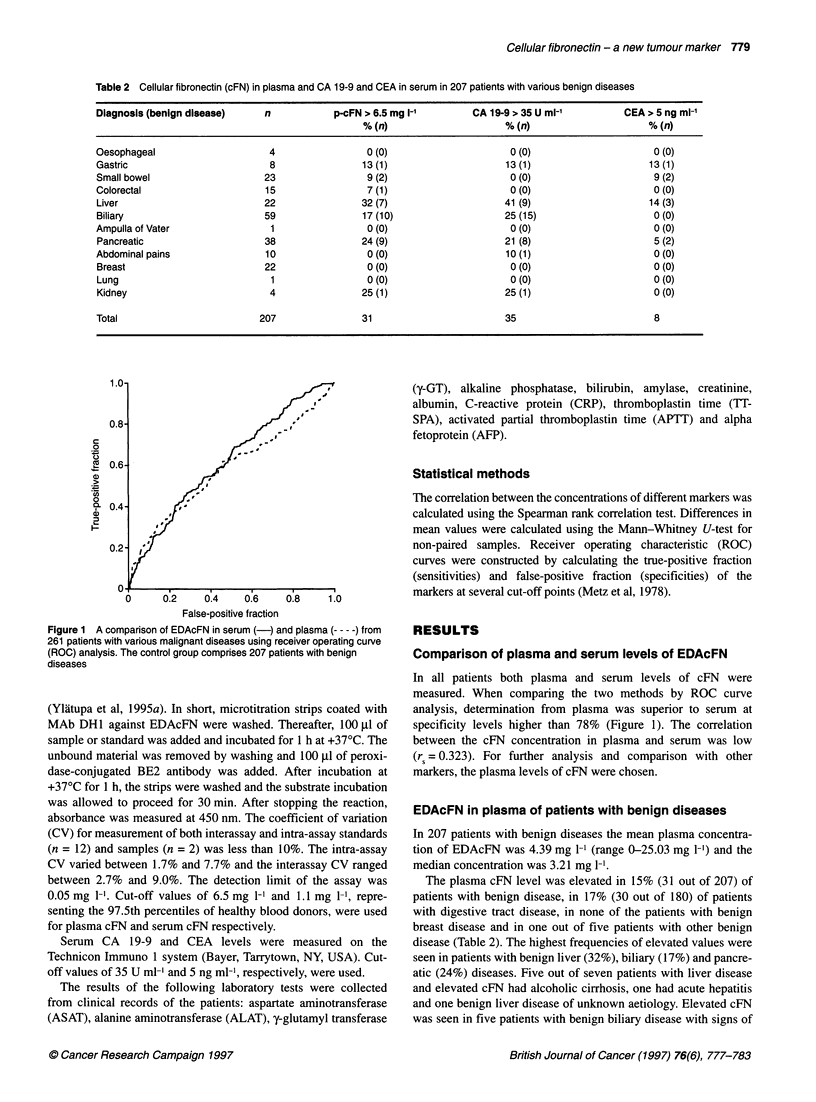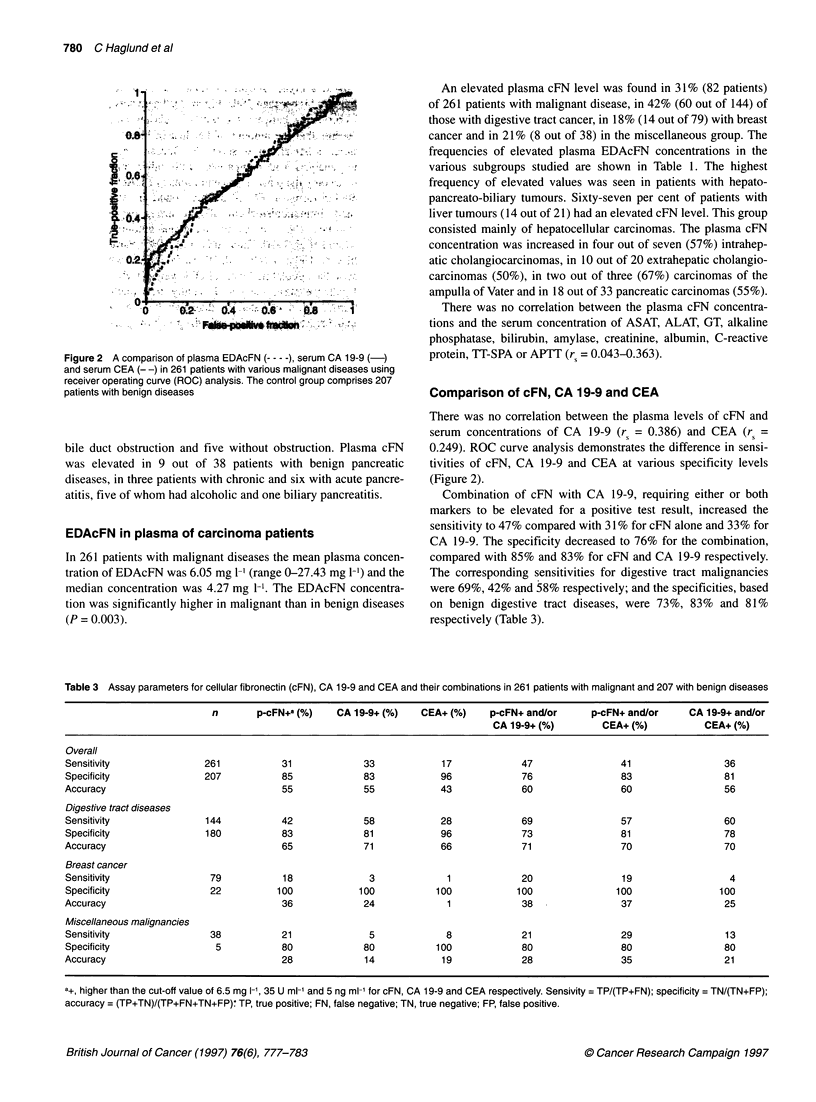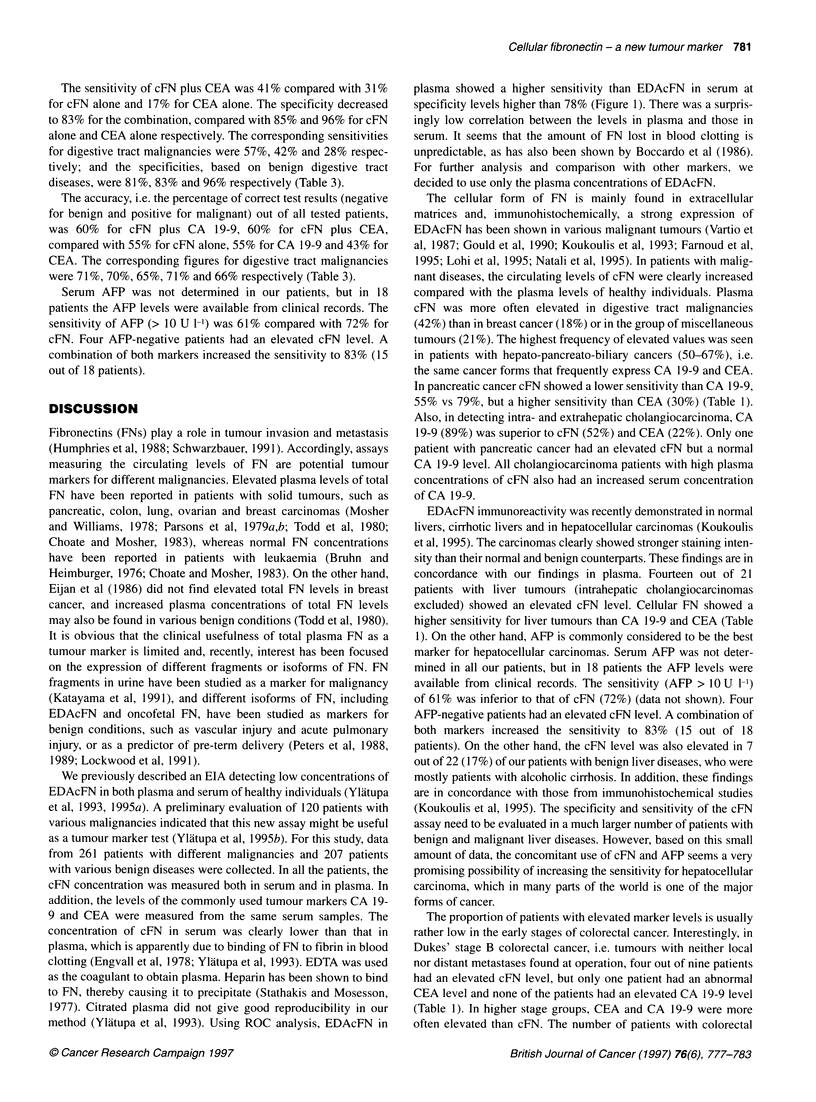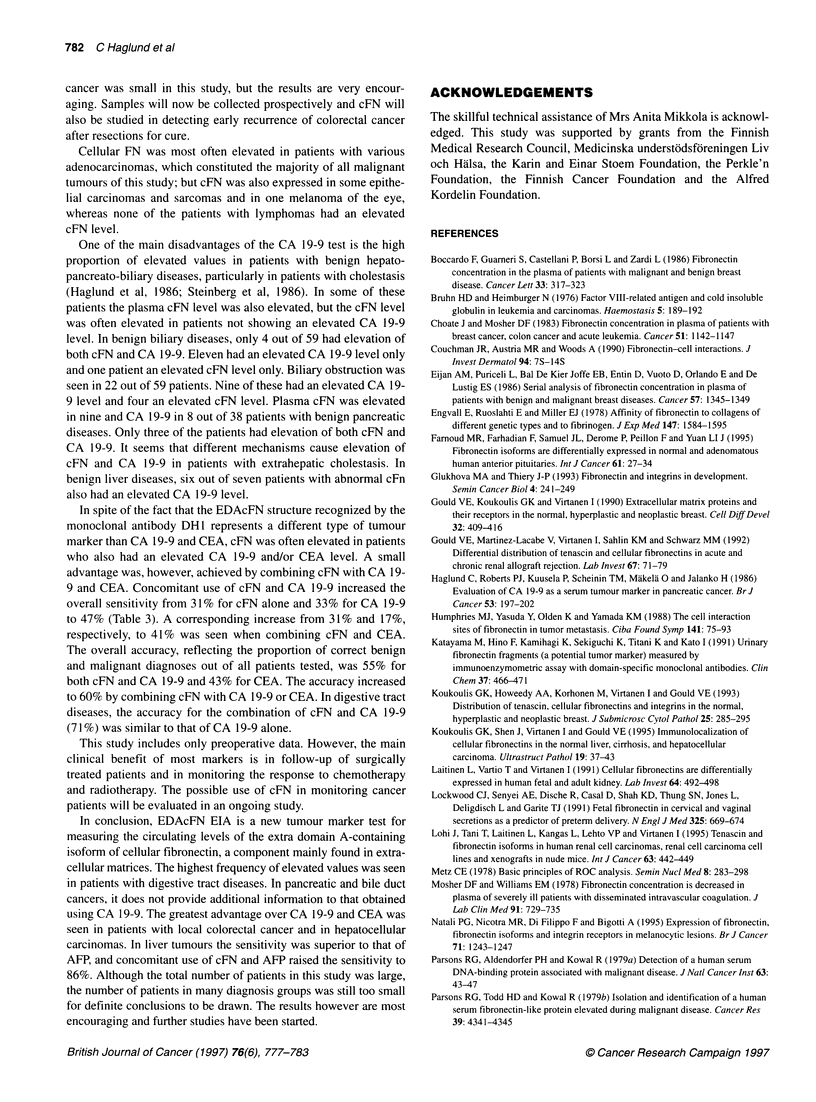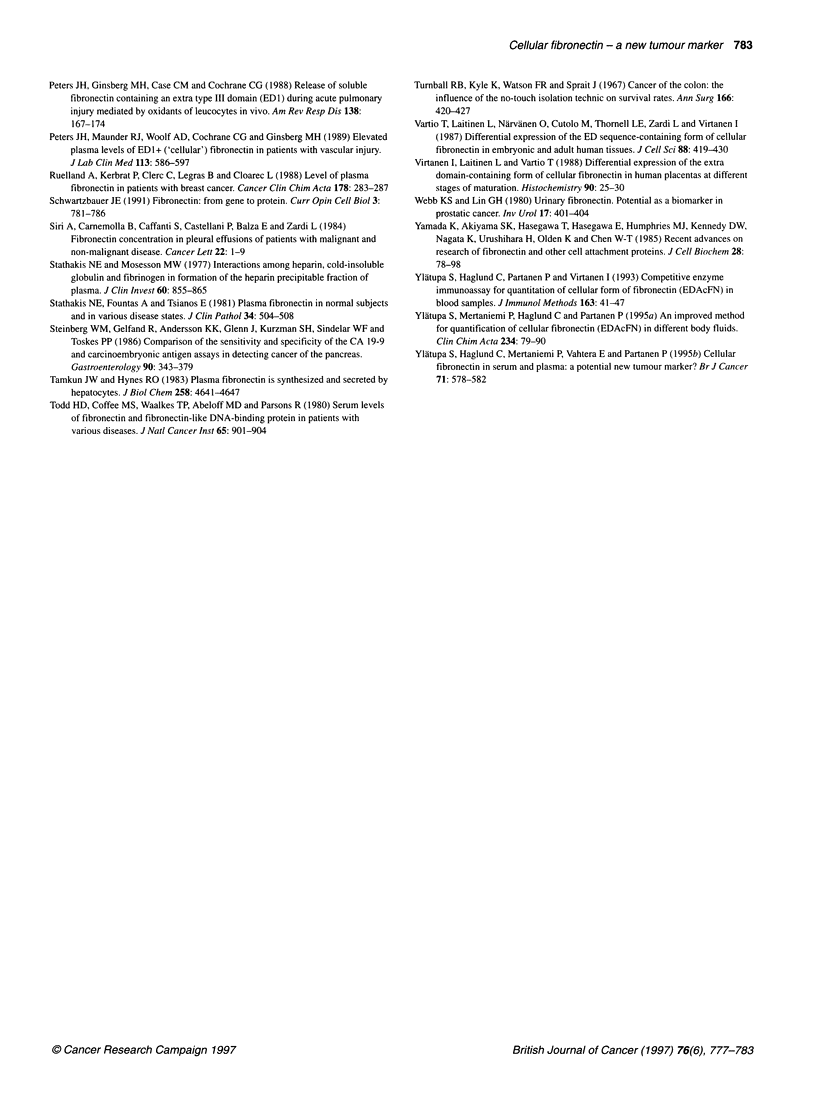# Cellular fibronectin concentration in the plasma of patients with malignant and benign diseases: a comparison with CA 19-9 and CEA.

**DOI:** 10.1038/bjc.1997.461

**Published:** 1997

**Authors:** C. Haglund, S. YlÃ¤tupa, P. Mertaniemi, P. Partanen

**Affiliations:** Department of Surgery, University of Helsinki, Finland.

## Abstract

EDAcFN enzyme immunoassay (EIA) is a new tumour marker assay measuring the extra domain A-containing isoform of cellular fibronectin (cFN), a component mainly found in extracellular matrices. The concentration cFN was measured in plasma and serum from 468 patients with malignant and benign diseases. The concentrations of cFN were higher in plasma than in serum. Using receiver operating characteristic (ROC) curve analysis, determination from plasma was superior to serum at specificity levels higher than 78% and was chosen for further analysis. The highest frequencies of elevated cFN values were seen in patients with hepato-pancreato-biliary malignancies (50-67%). In pancreatic and bile duct cancers, cFN provided little further information to that obtained by CA 19-9. The greatest advantage over CA 19-9 and CEA was seen in patients with local colorectal cancer and in hepatocellular carcinomas. Four out of nine patients with Dukes' stage B colorectal cancer had an elevated cFn level, but only one had an abnormal CEA level. In hepatocellular carcinomas, cFN was also compared with alpha-fetoprotein. The sensitivity of cFN (72%) was superior to that of AFP (61%), and concomitant use of cFN and AFP raised the sensitivity to 83%. The highest frequencies of elevated values in patients with benign diseases were observed in those with severe liver disease (32%) and biliary (17%) and pancreatic (24%) diseases. A combination of cFN and CA 19-9 showed the highest overall sensitivity of 47%, compared with 31% for cFN and 33% for CA 19-9. The corresponding specificities were 76% for cFN +/- CA 19-9, 85% for cFN and 83% for CA 19-9. The accuracy of a combination of cFN and CA 19-9 or CEA (60% respectively) was higher than that of cFN (55%), CA 19-9 (55%) or CEA (45%) alone. In conclusion, the results of the new cFN test are encouraging and further studies on larger patient materials have been started.


British Journal of Cancer (1997) 76(6), 777–783
© 1997 Cancer Research Campaign

### Cellular fibronectin concentration in the plasma of patients with malignant and benign diseases: a comparison with CA 19-9 and CEA

C Haglund[1], S Ylätupa[2], P Mertaniemi[2] and P Partanen[2]

[1]Department of Surgery, University of Helsinki; [2]Locus genex Oy, Helsinki, Finland

**Summary** EDAcFN enzyme immunoassay (EIA) is a new tumour marker assay measuring the extra domain A-containing isoform of cellular fibronectin (cFN), a component mainly found in extracellular matrices. The concentration cFN was measured in plasma and serum from 468 patients with malignant and benign diseases. The concentrations of cFN were higher in plasma than in serum. Using receiver operating characteristic (ROC) curve analysis, determination from plasma was superior to serum at specificity levels higher than 78% and was chosen for further analysis. The highest frequencies of elevated cFN values were seen in patients with hepato-pancreato-biliary malignancies (50–67%). In pancreatic and bile duct cancers, cFN provided little further information to that obtained by CA 19-9. The greatest advantage over CA 19-9 and CEA was seen in patients with local colorectal cancer and in hepatocellular carcinomas. Four out of nine patients with Dukes' stage B colorectal cancer had an elevated cFn level, but only one had an abnormal CEA level. In hepatocellular carcinomas, cFN was also compared with alpha-fetoprotein. The sensitivity of cFN (72%) was superior to that of AFP (61%), and concomitant use of cFN and AFP raised the sensitivity to 83%. The highest frequencies of elevated values in patients with benign diseases were observed in those with severe liver disease (32%) and biliary (17%) and pancreatic (24%) diseases. A combination of cFN and CA 19-9 showed the highest overall sensitivity of 47%, compared with 31% for cFN and 33% for CA 19-9. The corresponding specificities were 76% for cFN ± CA 19-9, 85% for cFN and 83% for CA 19-9. The accuracy of a combination of cFN and CA 19-9 or CEA (60% respectively) was higher than that of cFN (55%), CA 19-9 (55%) or CEA (45%) alone. In conclusion, the results of the new cFN test are encouraging and further studies on larger patient materials have been started.

**Keywords:** extradomain A; fibronectin; immunoassay; digestive tract neoplasm; CA 19-9; CEA; tumour marker

Fibronectins (FN) are adhesive glycoproteins that have variable primary structures owing to cell type-specific splicing of FN precursor mRNA. FNs can be divided into two major forms: plasma fibronectin (pFN), a soluble component of plasma and body fluids, and cellular fibronectin (cFN), mainly found in extracellular matrices. cFNs differ from pFN in having the so-called extra domain (ED) sequences A or B in the molecule (Schwarzbauer, 1991). Plasma FN is produced by hepatocytes, while cFNs are produced locally (Tamkun et al, 1983). However, plasma also contains small quantities of cFN (Vartio et al, 1987; Ylätupa et al, 1995*a*,*b*).

FNs have a role in various biological phenomena, such as tissue organization, cell adhesion, mobility and differentiation, as well as in tumour invasion and metastasis (Yamada et al, 1985; Humphries et al, 1988; Coachman et al, 1990; Schwarzbauer, 1991). In many studies, total FN in plasma and other body fluids has been evaluated as a marker for cancer or other diseases (Parsons et al, 1979*a*,*b*; Webb and Linn, 1980; Stathakis et al, 1981; Choate and Mosher, 1983; Siri et al, 1984; Boccardo et al, 1986; Ruelland et al, 1988; Katayama et al, 1991). Only recently have specific antibodies made it possible to study the cellular form of FN containing the EDA sequence (EDAcFN). In immunohistochemical stainings, EDAcFN has been shown to be present in abundance in certain developing basement membranes and in reactive adult tissues (Vartio et al, 1987; Virtanen et al, 1988; Gould et al, 1990, 1992; Laitinen et al, 1991; Glukhova and Thiery, 1993; Koukoulis et al, 1993). EDAcFN also showed a strong expression in the stroma of all carcinomas studied by Vartio et al (1987). A quantitative enzyme immunoassay based on the monoclonal antibody (MAb) DH1 detecting the EDAcFN has been described (Ylätupa et al, 1993, 1995*a*). In a recent report, cFN in plasma and serum was shown to be a promising tumour marker (Ylätupa et al, 1995*b*). In this study, data from patients with various malignant and benign diseases are reported. The results of cFN are compared with those of CA 19-9 and CEA, two widely used markers in clinical practice.

*Received 24 April 1996*
*Revised 7 February 1997*
*Accepted 20 February 1997*

*Correspondence to:* Caj Haglund, Department of Surgery, Helsinki University Central Hospital, Haartmaninkatu 4, FIN-00290 Helsinki

### MATERIALS AND METHODS

### Serum and plasma samples

Serum samples were obtained from 261 patients with different malignancies and from 207 patients with various benign diseases. Blood was collected by venepuncture into sodium EDTA (final concentration 4 mmol $l^{-1}$). Plasma was separated by centrifugation at 1400 *g* at room temperature. Blood for serum samples was allowed to coagulate at +4°C for 1 h before separation by centrifugation. Samples were stored at –70°C and thawed at +4°C for 12 h before the assay. In patients with recurrent colorectal carcinoma, the samples were taken at the time of clinical verification. In all other cancer patients, the samples were taken before surgical

Table 1 Cellular fibronectin (cFN) in plasma and CA 19-9 and CEA in serum in 261 patients with various malignant diseases

| Diagnosis (malignancy) | n | p-cFN > 6.5 mg l$^{-1}$ % (n) | CA 19-9 > 35 U ml$^{-1}$ % (n) | CEA > 5 ng ml$^{-1}$ % (n) |
|---|---|---|---|---|
| Oesophageal | 4 | 50 (2) | 0 (0) | 0 (0) |
| Gastric | 18 | 17 (3) | 44 (8) | 22 (4) |
| Stage I | 2 | (1) | (0) | (0) |
| Stage II | 6 | (0) | (1) | (0) |
| Stage III | 2 | (0) | (1) | (1) |
| Stage IV | 8 | (2) | (6) | (3) |
| Small bowel | 3 | 0 (0) | 33 (1) | 33 (1) |
| Colorectal | 35 | 20 (7) | 37 (13) | 49 (17) |
| Dukes stage B | 9 | (4) | (0) | (1) |
| Dukes stage C | 5 | (0) | (2) | (2) |
| Dukes stage D | 6 | (1) | (4) | (5) |
| Recurrent disease | 14 | (2) | (7) | (9) |
| Liver (hepatocellular) | 21 | 67 (14) | 43 (9) | 14 (3) |
| Stage III | 8 | (4) | (2) | (0) |
| Stage IV | 13 | (10) | (7) | (3) |
| Liver (bile duct) | 7 | 57 (4) | 100 (7) | 43 (3) |
| Stage III | 2 | (1) | (2) | (1) |
| Stage IV | 5 | (3) | (5) | (2) |
| Biliary (extrahepatic) | 20 | 50 (10) | 85 (17) | 15 (3) |
| Stage II | 2 | (2) | (2) | (0) |
| Stage III | 5 | (1) | (4) | (0) |
| Stage IV | 13 | (7) | (11) | (3) |
| Ampulla of Vater | 3 | 67 (2) | 67 (2) | 0 (0) |
| Pancreatic | 33 | 55 (18) | 79 (26) | 30 (10) |
| Stage I | 2 | (0) | (0) | (0) |
| Stage II | 11 | (8) | (9) | (2) |
| Stage III | 10 | (4) | (7) | (2) |
| Stage IV | 10 | (6) | (10) | (6) |
| Breast | 79 | 18 (14) | 3 (2) | 1 (1) |
| Stage 0 (in situ) | 3 | (0) | (0) | (0) |
| Stage I | 33 | (8) | (1) | (0) |
| Stage II | 36 | (5) | (1) | (1) |
| Stage III | 7 | (1) | (0) | (0) |
| Sarcoma | 10 | 20 (2) | 10 (1) | 0 (0) |
| Lung | 9 | 11 (1) | 0 (0) | 33 (3) |
| Urological | 10 | 40 (4) | 10 (1) | 0 (0) |
| Melanoma | 2 | 50 (1) | 0 (0) | 0 (0) |
| Thyroid | 1 | 0 (0) | 0 (0) | 0 (0) |
| Lymphoma | 6 | 0 (0) | 0 (0) | 0 (0) |
| Total | 261 | 82 | 87 | 45 |

therapy. Patients receiving chemotherapy or radiotherapy were not included in the study. The diagnoses were based on histological or cytological data and on clinical and laboratory findings. Patients with malignant tumours were divided into three groups: 144 patients with digestive tract malignancy (four oesophageal, 18 gastric, three small bowel, 35 colorectal, 33 pancreatic, 21 liver, seven intrahepatic biliary, 20 extrahepatic biliary and three ampulla of Vater), 79 patients with breast cancer and 38 patients with miscellaneous malignancies (two urinary bladder, six renal, two prostatic, nine lung, one thyroid, ten sarcomas, six lymphomas and one eye melanoma) (Table 1).

The group of benign diseases comprised 180 patients with benign digestive tract diseases (four oesophageal, eight gastric, 23 small bowel, 15 colorectal, 22 liver, 59 biliary, one ampulla of Vater, 38 pancreatic and ten patients with abdominal pains of unknown origin), 22 with benign breast diseases, one benign lung and four with renal insufficiency (Table 2).

Cancer patients were classified according to the UICC TNM classification, except for patients with colorectal cancer for whom the modified Dukes' classification was used (Turnball et al, 1967).

This study was carried out with ethical committee approval.

### Assays

The concentration of EDAcFN in serum and plasma samples was measured using an enzyme immunoassay as described previously

 © Cancer Research Campaign 1997

Table 2 Cellular fibronectin (cFN) in plasma and CA 19-9 and CEA in serum in 207 patients with various benign diseases

| Diagnosis (benign disease) | n | p-cFN > 6.5 mg $l^{-1}$ % (n) | CA 19-9 > 35 U $ml^{-1}$ % (n) | CEA > 5 ng $ml^{-1}$ % (n) |
|---|---|---|---|---|
| Oesophageal | 4 | 0 (0) | 0 (0) | 0 (0) |
| Gastric | 8 | 13 (1) | 13 (1) | 13 (1) |
| Small bowel | 23 | 9 (2) | 0 (0) | 9 (2) |
| Colorectal | 15 | 7 (1) | 0 (0) | 0 (0) |
| Liver | 22 | 32 (7) | 41 (9) | 14 (3) |
| Biliary | 59 | 17 (10) | 25 (15) | 0 (0) |
| Ampulla of Vater | 1 | 0 (0) | 0 (0) | 0 (0) |
| Pancreatic | 38 | 24 (9) | 21 (8) | 5 (2) |
| Abdominal pains | 10 | 0 (0) | 10 (1) | 0 (0) |
| Breast | 22 | 0 (0) | 0 (0) | 0 (0) |
| Lung | 1 | 0 (0) | 0 (0) | 0 (0) |
| Kidney | 4 | 25 (1) | 25 (1) | 0 (0) |
| Total | 207 | 31 | 35 | 8 |

Figure 1 A comparison of EDAcFN in serum (—) and plasma (- - - -) from 261 patients with various malignant diseases using receiver operating curve (ROC) analysis. The control group comprises 207 patients with benign diseases

(Ylätupa et al, 1995*a*). In short, microtitration strips coated with MAb DH1 against EDAcFN were washed. Thereafter, 100 μl of sample or standard was added and incubated for 1 h at +37°C. The unbound material was removed by washing and 100 μl of peroxidase-conjugated BE2 antibody was added. After incubation at +37°C for 1 h, the strips were washed and the substrate incubation was allowed to proceed for 30 min. After stopping the reaction, absorbance was measured at 450 nm. The coefficient of variation (CV) for measurement of both interassay and intra-assay standards ($n$ = 12) and samples ($n$ = 2) was less than 10%. The intra-assay CV varied between 1.7% and 7.7% and the interassay CV ranged between 2.7% and 9.0%. The detection limit of the assay was 0.05 mg $l^{-1}$. Cut-off values of 6.5 mg $l^{-1}$ and 1.1 mg $l^{-1}$, representing the 97.5th percentiles of healthy blood donors, were used for plasma cFN and serum cFN respectively.

Serum CA 19-9 and CEA levels were measured on the Technicon Immuno 1 system (Bayer, Tarrytown, NY, USA). Cut-off values of 35 U $ml^{-1}$ and 5 ng $ml^{-1}$, respectively, were used.

The results of the following laboratory tests were collected from clinical records of the patients: aspartate aminotransferase (ASAT), alanine aminotransferase (ALAT), γ-glutamyl transferase (γ-GT), alkaline phosphatase, bilirubin, amylase, creatinine, albumin, C-reactive protein (CRP), thromboplastin time (TT-SPA), activated partial thromboplastin time (APTT) and alpha fetoprotein (AFP).

### Statistical methods

The correlation between the concentrations of different markers was calculated using the Spearman rank correlation test. Differences in mean values were calculated using the Mann–Whitney *U*-test for non-paired samples. Receiver operating characteristic (ROC) curves were constructed by calculating the true-positive fraction (sensitivities) and false-positive fraction (specificities) of the markers at several cut-off points (Metz et al, 1978).

### RESULTS

### Comparison of plasma and serum levels of EDAcFN

In all patients both plasma and serum levels of cFN were measured. When comparing the two methods by ROC curve analysis, determination from plasma was superior to serum at specificity levels higher than 78% (Figure 1). The correlation between the cFN concentration in plasma and serum was low ($r_s$ = 0.323). For further analysis and comparison with other markers, the plasma levels of cFN were chosen.

### EDAcFN in plasma of patients with benign diseases

In 207 patients with benign diseases the mean plasma concentration of EDAcFN was 4.39 mg $l^{-1}$ (range 0–25.03 mg $l^{-1}$) and the median concentration was 3.21 mg $l^{-1}$.

The plasma cFN level was elevated in 15% (31 out of 207) of patients with benign disease, in 17% (30 out of 180) of patients with digestive tract disease, in none of the patients with benign breast disease and in one out of five patients with other benign disease (Table 2). The highest frequencies of elevated values were seen in patients with benign liver (32%), biliary (17%) and pancreatic (24%) diseases. Five out of seven patients with liver disease and elevated cFN had alcoholic cirrhosis, one had acute hepatitis and one benign liver disease of unknown aetiology. Elevated cFN was seen in five patients with benign biliary disease with signs of

© Cancer Research Campaign 1997

**Figure 2** A comparison of plasma EDAcFN (- - - -), serum CA 19-9 (—) and serum CEA (– –) in 261 patients with various malignant diseases using receiver operating curve (ROC) analysis. The control group comprises 207 patients with benign diseases bile duct obstruction and five without obstruction. Plasma cFN was elevated in 9 out of 38 patients with benign pancreatic diseases, in three patients with chronic and six with acute pancreatitis, five of whom had alcoholic and one biliary pancreatitis.

### EDAcFN in plasma of carcinoma patients

In 261 patients with malignant diseases the mean plasma concentration of EDAcFN was 6.05 mg $l^{-1}$ (range 0–27.43 mg $l^{-1}$) and the median concentration was 4.27 mg $l^{-1}$. The EDAcFN concentration was significantly higher in malignant than in benign diseases ($P = 0.003$).

An elevated plasma cFN level was found in 31% (82 patients) of 261 patients with malignant disease, in 42% (60 out of 144) of those with digestive tract cancer, in 18% (14 out of 79) with breast cancer and in 21% (8 out of 38) in the miscellaneous group. The frequencies of elevated plasma EDAcFN concentrations in the various subgroups studied are shown in Table 1. The highest frequency of elevated values was seen in patients with hepato-pancreato-biliary tumours. Sixty-seven per cent of patients with liver tumours (14 out of 21) had an elevated cFN level. This group consisted mainly of hepatocellular carcinomas. The plasma cFN concentration was increased in four out of seven (57%) intrahepatic cholangiocarcinomas, in 10 out of 20 extrahepatic cholangiocarcinomas (50%), in two out of three (67%) carcinomas of the ampulla of Vater and in 18 out of 33 pancreatic carcinomas (55%).

There was no correlation between the plasma cFN concentrations and the serum concentration of ASAT, ALAT, GT, alkaline phosphatase, bilirubin, amylase, creatinine, albumin, C-reactive protein, TT-SPA or APTT ($r_s = 0.043–0.363$).

### Comparison of cFN, CA 19-9 and CEA

There was no correlation between the plasma levels of cFN and serum concentrations of CA 19-9 ($r_s = 0.386$) and CEA ($r_s = 0.249$). ROC curve analysis demonstrates the difference in sensitivities of cFN, CA 19-9 and CEA at various specificity levels (Figure 2).

Combination of cFN with CA 19-9, requiring either or both markers to be elevated for a positive test result, increased the sensitivity to 47% compared with 31% for cFN alone and 33% for CA 19-9. The specificity decreased to 76% for the combination, compared with 85% and 83% for cFN and CA 19-9 respectively. The corresponding sensitivities for digestive tract malignancies were 69%, 42% and 58% respectively; and the specificities, based on benign digestive tract diseases, were 73%, 83% and 81% respectively (Table 3).

**Table 3** Assay parameters for cellular fibronectin (cFN), CA 19-9 and CEA and their combinations in 261 patients with malignant and 207 with benign diseases

| | *n* | p-cFN+[a] (%) | CA 19-9+ (%) | CEA+ (%) | p-cFN+ and/or CA 19-9+ (%) | p-cFN+ and/or CEA+ (%) | CA 19-9+ and/or CEA+ (%) |
|---|---|---|---|---|---|---|---|
| *Overall* | | | | | | | |
| Sensitivity | 261 | 31 | 33 | 17 | 47 | 41 | 36 |
| Specificity | 207 | 85 | 83 | 96 | 76 | 83 | 81 |
| Accuracy | | 55 | 55 | 43 | 60 | 60 | 56 |
| *Digestive tract diseases* | | | | | | | |
| Sensitivity | 144 | 42 | 58 | 28 | 69 | 57 | 60 |
| Specificity | 180 | 83 | 81 | 96 | 73 | 81 | 78 |
| Accuracy | | 65 | 71 | 66 | 71 | 70 | 70 |
| *Breast cancer* | | | | | | | |
| Sensitivity | 79 | 18 | 3 | 1 | 20 | 19 | 4 |
| Specificity | 22 | 100 | 100 | 100 | 100 | 100 | 100 |
| Accuracy | | 36 | 24 | 1 | 38 | 37 | 25 |
| *Miscellaneous malignancies* | | | | | | | |
| Sensitivity | 38 | 21 | 5 | 8 | 21 | 29 | 13 |
| Specificity | 5 | 80 | 80 | 100 | 80 | 80 | 80 |
| Accuracy | | 28 | 14 | 19 | 28 | 35 | 21 |

[a]+, higher than the cut-off value of 6.5 mg $l^{-1}$, 35 U $ml^{-1}$ and 5 ng $ml^{-1}$ for cFN, CA 19-9 and CEA respectively. Sensivity = TP/(TP+FN); specificity = TN/(TN+FP); accuracy = (TP+TN)/(TP+FN+TN+FP): TP, true positive; FN, false negative; TN, true negative; FP, false positive.

 © Cancer Research Campaign 1997

The sensitivity of cFN plus CEA was 41% compared with 31% for cFN alone and 17% for CEA alone. The specificity decreased to 83% for the combination, compared with 85% and 96% for cFN alone and CEA alone respectively. The corresponding sensitivities for digestive tract malignancies were 57%, 42% and 28% respectively; and the specificities, based on benign digestive tract diseases, were 81%, 83% and 96% respectively (Table 3).

The accuracy, i.e. the percentage of correct test results (negative for benign and positive for malignant) out of all tested patients, was 60% for cFN plus CA 19-9, 60% for cFN plus CEA, compared with 55% for cFN alone, 55% for CA 19-9 and 43% for CEA. The corresponding figures for digestive tract malignancies were 71%, 70%, 65%, 71% and 66% respectively (Table 3).

Serum AFP was not determined in our patients, but in 18 patients the AFP levels were available from clinical records. The sensitivity of AFP (> 10 U $l^{-1}$) was 61% compared with 72% for cFN. Four AFP-negative patients had an elevated cFN level. A combination of both markers increased the sensitivity to 83% (15 out of 18 patients).

### DISCUSSION

Fibronectins (FNs) play a role in tumour invasion and metastasis (Humphries et al, 1988; Schwarzbauer, 1991). Accordingly, assays measuring the circulating levels of FN are potential tumour markers for different malignancies. Elevated plasma levels of total FN have been reported in patients with solid tumours, such as pancreatic, colon, lung, ovarian and breast carcinomas (Mosher and Williams, 1978; Parsons et al, 1979*a*,*b*; Todd et al, 1980; Choate and Mosher, 1983), whereas normal FN concentrations have been reported in patients with leukaemia (Bruhn and Heimburger, 1976; Choate and Mosher, 1983). On the other hand, Eijan et al (1986) did not find elevated total FN levels in breast cancer, and increased plasma concentrations of total FN levels may also be found in various benign conditions (Todd et al, 1980). It is obvious that the clinical usefulness of total plasma FN as a tumour marker is limited and, recently, interest has been focused on the expression of different fragments or isoforms of FN. FN fragments in urine have been studied as a marker for malignancy (Katayama et al, 1991), and different isoforms of FN, including EDAcFN and oncofetal FN, have been studied as markers for benign conditions, such as vascular injury and acute pulmonary injury, or as a predictor of pre-term delivery (Peters et al, 1988, 1989; Lockwood et al, 1991).

We previously described an EIA detecting low concentrations of EDAcFN in both plasma and serum of healthy individuals (Ylätupa et al, 1993, 1995*a*). A preliminary evaluation of 120 patients with various malignancies indicated that this new assay might be useful as a tumour marker test (Ylätupa et al, 1995*b*). For this study, data from 261 patients with different malignancies and 207 patients with various benign diseases were collected. In all the patients, the cFN concentration was measured both in serum and in plasma. In addition, the levels of the commonly used tumour markers CA 19-9 and CEA were measured from the same serum samples. The concentration of cFN in serum was clearly lower than that in plasma, which is apparently due to binding of FN to fibrin in blood clotting (Engvall et al, 1978; Ylätupa et al, 1993). EDTA was used as the coagulant to obtain plasma. Heparin has been shown to bind to FN, thereby causing it to precipitate (Stathakis and Mosesson, 1977). Citrated plasma did not give good reproducibility in our method (Ylätupa et al, 1993). Using ROC analysis, EDAcFN in plasma showed a higher sensitivity than EDAcFN in serum at specificity levels higher than 78% (Figure 1). There was a surprisingly low correlation between the levels in plasma and those in serum. It seems that the amount of FN lost in blood clotting is unpredictable, as has also been shown by Boccardo et al (1986). For further analysis and comparison with other markers, we decided to use only the plasma concentrations of EDAcFN.

The cellular form of FN is mainly found in extracellular matrices and, immunohistochemically, a strong expression of EDAcFN has been shown in various malignant tumours (Vartio et al, 1987; Gould et al, 1990; Koukoulis et al, 1993; Farnoud et al, 1995; Lohi et al, 1995; Natali et al, 1995). In patients with malignant diseases, the circulating levels of cFN were clearly increased compared with the plasma levels of healthy individuals. Plasma cFN was more often elevated in digestive tract malignancies (42%) than in breast cancer (18%) or in the group of miscellaneous tumours (21%). The highest frequency of elevated values was seen in patients with hepato-pancreato-biliary cancers (50–67%), i.e. the same cancer forms that frequently express CA 19-9 and CEA. In pancreatic cancer cFN showed a lower sensitivity than CA 19-9, 55% vs 79%, but a higher sensitivity than CEA (30%) (Table 1). Also, in detecting intra- and extrahepatic cholangiocarcinoma, CA 19-9 (89%) was superior to cFN (52%) and CEA (22%). Only one patient with pancreatic cancer had an elevated cFN but a normal CA 19-9 level. All cholangiocarcinoma patients with high plasma concentrations of cFN also had an increased serum concentration of CA 19-9.

EDAcFN immunoreactivity was recently demonstrated in normal livers, cirrhotic livers and in hepatocellular carcinomas (Koukoulis et al, 1995). The carcinomas clearly showed stronger staining intensity than their normal and benign counterparts. These findings are in concordance with our findings in plasma. Fourteen out of 21 patients with liver tumours (intrahepatic cholangiocarcinomas excluded) showed an elevated cFN level. Cellular FN showed a higher sensitivity for liver tumours than CA 19-9 and CEA (Table 1). On the other hand, AFP is commonly considered to be the best marker for hepatocellular carcinomas. Serum AFP was not determined in all our patients, but in 18 patients the AFP levels were available from clinical records. The sensitivity (AFP > 10 U $l^{-1}$) of 61% was inferior to that of cFN (72%) (data not shown). Four AFP-negative patients had an elevated cFN level. A combination of both markers increased the sensitivity to 83% (15 out of 18 patients). On the other hand, the cFN level was also elevated in 7 out of 22 (17%) of our patients with benign liver diseases, who were mostly patients with alcoholic cirrhosis. In addition, these findings are in concordance with those from immunohistochemical studies (Koukoulis et al, 1995). The specificity and sensitivity of the cFN assay need to be evaluated in a much larger number of patients with benign and malignant liver diseases. However, based on this small amount of data, the concomitant use of cFN and AFP seems a very promising possibility of increasing the sensitivity for hepatocellular carcinoma, which in many parts of the world is one of the major forms of cancer.

The proportion of patients with elevated marker levels is usually rather low in the early stages of colorectal cancer. Interestingly, in Dukes' stage B colorectal cancer, i.e. tumours with neither local nor distant metastases found at operation, four out of nine patients had an elevated cFN level, but only one patient had an abnormal CEA level and none of the patients had an elevated CA 19-9 level (Table 1). In higher stage groups, CEA and CA 19-9 were more often elevated than cFN. The number of patients with colorectal

© Cancer Research Campaign 1997

cancer was small in this study, but the results are very encouraging. Samples will now be collected prospectively and cFN will also be studied in detecting early recurrence of colorectal cancer after resections for cure.

Cellular FN was most often elevated in patients with various adenocarcinomas, which constituted the majority of all malignant tumours of this study; but cFN was also expressed in some epithelial carcinomas and sarcomas and in one melanoma of the eye, whereas none of the patients with lymphomas had an elevated cFN level.

One of the main disadvantages of the CA 19-9 test is the high proportion of elevated values in patients with benign hepato-pancreato-biliary diseases, particularly in patients with cholestasis (Haglund et al, 1986; Steinberg et al, 1986). In some of these patients the plasma cFN level was also elevated, but the cFN level was often elevated in patients not showing an elevated CA 19-9 level. In benign biliary diseases, only 4 out of 59 had elevation of both cFN and CA 19-9. Eleven had an elevated CA 19-9 level only and one patient an elevated cFN level only. Biliary obstruction was seen in 22 out of 59 patients. Nine of these had an elevated CA 19-9 level and four an elevated cFN level. Plasma cFN was elevated in nine and CA 19-9 in 8 out of 38 patients with benign pancreatic diseases. Only three of the patients had elevation of both cFN and CA 19-9. It seems that different mechanisms cause elevation of cFN and CA 19-9 in patients with extrahepatic cholestasis. In benign liver diseases, six out of seven patients with abnormal cFn also had an elevated CA 19-9 level.

In spite of the fact that the EDAcFN structure recognized by the monoclonal antibody DH1 represents a different type of tumour marker than CA 19-9 and CEA, cFN was often elevated in patients who also had an elevated CA 19-9 and/or CEA level. A small advantage was, however, achieved by combining cFN with CA 19-9 and CEA. Concomitant use of cFN and CA 19-9 increased the overall sensitivity from 31% for cFN alone and 33% for CA 19-9 to 47% (Table 3). A corresponding increase from 31% and 17%, respectively, to 41% was seen when combining cFN and CEA. The overall accuracy, reflecting the proportion of correct benign and malignant diagnoses out of all patients tested, was 55% for both cFN and CA 19-9 and 43% for CEA. The accuracy increased to 60% by combining cFN with CA 19-9 or CEA. In digestive tract diseases, the accuracy for the combination of cFN and CA 19-9 (71%) was similar to that of CA 19-9 alone.

This study includes only preoperative data. However, the main clinical benefit of most markers is in follow-up of surgically treated patients and in monitoring the response to chemotherapy and radiotherapy. The possible use of cFN in monitoring cancer patients will be evaluated in an ongoing study.

In conclusion, EDAcFN EIA is a new tumour marker test for measuring the circulating levels of the extra domain A-containing isoform of cellular fibronectin, a component mainly found in extracellular matrices. The highest frequency of elevated values was seen in patients with digestive tract diseases. In pancreatic and bile duct cancers, it does not provide additional information to that obtained using CA 19-9. The greatest advantage over CA 19-9 and CEA was seen in patients with local colorectal cancer and in hepatocellular carcinomas. In liver tumours the sensitivity was superior to that of AFP, and concomitant use of cFN and AFP raised the sensitivity to 86%. Although the total number of patients in this study was large, the number of patients in many diagnosis groups was still too small for definite conclusions to be drawn. The results however are most encouraging and further studies have been started.

### ACKNOWLEDGEMENTS

The skillful technical assistance of Mrs Anita Mikkola is acknowledged. This study was supported by grants from the Finnish Medical Research Council, Medicinska understödsföreningen Liv och Hälsa, the Karin and Einar Stoem Foundation, the Perkle'n Foundation, the Finnish Cancer Foundation and the Alfred Kordelin Foundation.

### REFERENCES

Boccardo F, Guarneri S, Castellani P, Borsi L and Zardi L (1986) Fibronectin concentration in the plasma of patients with malignant and benign breast disease. *Cancer Lett* **33**: 317–323

Bruhn HD and Heimburger N (1976) Factor VIII-related antigen and cold insoluble globulin in leukemia and carcinomas. *Haemostasis* **5**: 189–192

Choate J and Mosher DF (1983) Fibronectin concentration in plasma of patients with breast cancer, colon cancer and acute leukemia. *Cancer* **51**: 1142–1147

Couchman JR, Austria MR and Woods A (1990) Fibronectin–cell interactions. *J Invest Dermatol* **94**: 7S–14S

Eijan AM, Puriceli L, Bal De Kier Joffe EB, Entin D, Vuoto D, Orlando E and De Lustig ES (1986) Serial analysis of fibronectin concentration in plasma of patients with benign and malignant breast diseases. *Cancer* **57**: 1345–1349

Engvall E, Ruoslahti E and Miller EJ (1978) Affinity of fibronectin to collagens of different genetic types and to fibrinogen. *J Exp Med* **147**: 1584–1595

Farnoud MR, Farhadian F, Samuel JL, Derome P, Peillon F and Yuan LI J (1995) Fibronectin isoforms are differentially expressed in normal and adenomatous human anterior pituitaries. *Int J Cancer* **61**: 27–34

Glukhova MA and Thiery J-P (1993) Fibronectin and integrins in development. *Semin Cancer Biol* **4**: 241–249

Gould VE, Koukoulis GK and Virtanen I (1990) Extracellular matrix proteins and their receptors in the normal, hyperplastic and neoplastic breast. *Cell Diff Devel* **32**: 409–416

Gould VE, Martinez-Lacabe V, Virtanen I, Sahlin KM and Schwarz MM (1992) Differential distribution of tenascin and cellular fibronectins in acute and chronic renal allograft rejection. *Lab Invest* **67**: 71–79

Haglund C, Roberts PJ, Kuusela P, Scheinin TM, Mäkelä O and Jalanko H (1986) Evaluation of CA 19-9 as a serum tumour marker in pancreatic cancer. *Br J Cancer* **53**: 197–202

Humphries MJ, Yasuda Y, Olden K and Yamada KM (1988) The cell interaction sites of fibronectin in tumor metastasis. *Ciba Found Symp* **141**: 75–93

Katayama M, Hino F, Kamihagi K, Sekiguchi K, Titani K and Kato I (1991) Urinary fibronectin fragments (a potential tumor marker) measured by immunoenzymometric assay with domain-specific monoclonal antibodies. *Clin Chem* **37**: 466–471

Koukoulis GK, Howeedy AA, Korhonen M, Virtanen I and Gould VE (1993) Distribution of tenascin, cellular fibronectins and integrins in the normal, hyperplastic and neoplastic breast. *J Submicrosc Cytol Pathol* **25**: 285–295

Koukoulis GK, Shen J, Virtanen I and Gould VE (1995) Immunolocalization of cellular fibronectins in the normal liver, cirrhosis, and hepatocellular carcinoma. *Ultrastruct Pathol* **19**: 37–43

Laitinen L, Vartio T and Virtanen I (1991) Cellular fibronectins are differentially expressed in human fetal and adult kidney. *Lab Invest* **64**: 492–498

Lockwood CJ, Senyei AE, Dische R, Casal D, Shah KD, Thung SN, Jones L, Deligdisch L and Garite TJ (1991) Fetal fibronectin in cervical and vaginal secretions as a predictor of preterm delivery. *N Engl J Med* **325**: 669–674

Lohi J, Tani T, Laitinen L, Kangas L, Lehto VP and Virtanen I (1995) Tenascin and fibronectin isoforms in human renal cell carcinomas, renal cell carcinoma cell lines and xenografts in nude mice. *Int J Cancer* **63**: 442–449

Metz CE (1978) Basic principles of ROC analysis. *Semin Nucl Med* **8**: 283–298

Mosher DF and Williams EM (1978) Fibronectin concentration is decreased in plasma of severely ill patients with disseminated intravascular coagulation. *J Lab Clin Med* **91**: 729–735

Natali PG, Nicotra MR, Di Filippo F and Bigotti A (1995) Expression of fibronectin, fibronectin isoforms and integrin receptors in melanocytic lesions. *Br J Cancer* **71**: 1243–1247

Parsons RG, Aldendorfer PH and Kowal R (1979*a*) Detection of a human serum DNA-binding protein associated with malignant disease. *J Natl Cancer Inst* **63**: 43–47

Parsons RG, Todd HD and Kowal R (1979*b*) Isolation and identification of a human serum fibronectin-like protein elevated during malignant disease. *Cancer Res* **39**: 4341–4345

© Cancer Research Campaign 1997

Peters JH, Ginsberg MH, Case CM and Cochrane CG (1988) Release of soluble fibronectin containing an extra type III domain (ED1) during acute pulmonary injury mediated by oxidants of leucocytes in vivo. *Am Rev Resp Dis* **138**: 167–174

Peters JH, Maunder RJ, Woolf AD, Cochrane CG and Ginsberg MH (1989) Elevated plasma levels of ED1+ ('cellular') fibronectin in patients with vascular injury. *J Lab Clin Med* **113**: 586–597

Ruelland A, Kerbrat P, Clerc C, Legras B and Cloarec L (1988) Level of plasma fibronectin in patients with breast cancer. *Cancer Clin Chim Acta* **178**: 283–287

Schwartzbauer JE (1991) Fibronectin: from gene to protein. *Curr Opin Cell Biol* **3**: 781–786

Siri A, Carnemolla B, Caffanti S, Castellani P, Balza E and Zardi L (1984) Fibronectin concentration in pleural effusions of patients with malignant and non-malignant disease. *Cancer Lett* **22**: 1–9

Stathakis NE and Mosesson MW (1977) Interactions among heparin, cold-insoluble globulin and fibrinogen in formation of the heparin precipitable fraction of plasma. *J Clin Invest* **60**: 855–865

Stathakis NE, Fountas A and Tsianos E (1981) Plasma fibronectin in normal subjects and in various disease states. *J Clin Pathol* **34**: 504–508

Steinberg WM, Gelfand R, Andersson KK, Glenn J, Kurzman SH, Sindelar WF and Toskes PP (1986) Comparison of the sensitivity and specificity of the CA 19-9 and carcinoembryonic antigen assays in detecting cancer of the pancreas. *Gastroenterology* **90**: 343–379

Tamkun JW and Hynes RO (1983) Plasma fibronectin is synthesized and secreted by hepatocytes. *J Biol Chem* **258**: 4641–4647

Todd HD, Coffee MS, Waalkes TP, Abeloff MD and Parsons R (1980) Serum levels of fibronectin and fibronectin-like DNA-binding protein in patients with various diseases. *J Natl Cancer Inst* **65**: 901–904

Turnball RB, Kyle K, Watson FR and Sprait J (1967) Cancer of the colon: the influence of the no-touch isolation technic on survival rates. *Ann Surg* **166**: 420–427

Vartio T, Laitinen L, Närvänen O, Cutolo M, Thornell LE, Zardi L and Virtanen I (1987) Differential expression of the ED sequence-containing form of cellular fibronectin in embryonic and adult human tissues. *J Cell Sci* **88**: 419–430

Virtanen I, Laitinen L and Vartio T (1988) Differential expression of the extra domain-containing form of cellular fibronectin in human placentas at different stages of maturation. *Histochemistry* **90**: 25–30

Webb KS and Lin GH (1980) Urinary fibronectin. Potential as a biomarker in prostatic cancer. *Inv Urol* **17**: 401–404

Yamada K, Akiyama SK, Hasegawa T, Hasegawa E, Humphries MJ, Kennedy DW, Nagata K, Urushihara H, Olden K and Chen W-T (1985) Recent advances on research of fibronectin and other cell attachment proteins. *J Cell Biochem* **28**: 78–98

Ylätupa S, Haglund C, Partanen P and Virtanen I (1993) Competitive enzyme immunoassay for quantitation of cellular form of fibronectin (EDAcFN) in blood samples. *J Immunol Methods* **163**: 41–47

Ylätupa S, Mertaniemi P, Haglund C and Partanen P (1995*a*) An improved method for quantification of cellular fibronectin (EDAcFN) in different body fluids. *Clin Chim Acta* **234**: 79–90

Ylätupa S, Haglund C, Mertaniemi P, Vahtera E and Partanen P (1995*b*) Cellular fibronectin in serum and plasma: a potential new tumour marker? *Br J Cancer* **71**: 578–582

© Cancer Research Campaign 1997